# The Association Between Religion and Healthcare Professionals’ Attitudes Towards the Conscience Clause. A Preliminary Study From Poland

**DOI:** 10.3389/ijph.2023.1606526

**Published:** 2023-12-01

**Authors:** Justyna Czekajewska, Dariusz Walkowiak, Jan Domaradzki

**Affiliations:** ^1^ Department of Social Sciences and Humanities, Poznan University of Medical Sciences, Poznań, Poland; ^2^ Department of Organization and Management in Health Care, Poznan University of Medical Sciences, Poznań, Poland

**Keywords:** conscience clause, healthcare professionals, nurses, pharmacists, physicians, religion

## Abstract

**Objectives:** This study was designed to determine the relationship between religion and healthcare practitioners’ attitudes towards conscience clauses in Poland.

**Methods:** We developed a survey assessing impact of religion on attitudes healthcare professionals towards the conscience clause. These questions were explored using a sample of 300 Polish healthcare professionals.

**Results:** The results indicate that religiosity was a significant predictor of acceptance of conscience clauses. It also influenced healthcare practitioners’ opinions on medical professionals that should be granted the right to conscience clauses and medical services that may be denied on moral grounds. There was also a significant relationship between healthcare practitioners’ religiosity and their eagerness to use conscience clauses in a situation of moral conflict. Finally, religious healthcare practitioners were more concerned about the personal consequences of using this right in a medical environment.

**Conclusion:** This study shows that at the same time, both religious and non-religious healthcare professionals believed that the Polish regulations regarding conscience clause are unclear and inaccurate, therefore leading to misinterpretation and abuse regulation of law.

## Introduction

One of the pillars of democratic order is the freedom of thought, conscience and religion, guaranteed by international human rights documents [1–3] and national constitutions [4, 5]. Similarly, pursuant to art. 53, paragraph 1 of the *Constitution of the Republic of Poland* “Everyone shall be guaranteed freedom of conscience and religion.” This right includes the freedom to choose one’s religion or moral beliefs, the freedom to manifest them individually or with other people, privately or publicly, through worship, prayer, and participation in rituals [6].

Respect for the principle of religious diversity and pluralism of values leads to the idea that everyone has the right to choose any religious, moral or philosophical beliefs he or she identifies with. Problems arise, however, when personal moral beliefs influence social discourse on such bioethical topics as abortion, contraception or euthanasia, which, in turn, may cause moral conflicts among healthcare professionals (HCPs). This may, in turn, hinder the individual right to healthcare. Consequently, every time such issues emerge in public discourse in Poland they provoke a heated debate on the legal sanctioning of the conscience clause (CC) for various medical professions.

The CC itself is a legal norm that, under certain circumstances, allows selected healthcare professionals to refuse to provide a medical service that is permitted by law, but which offends their conscience [7]. It should be noted, however, that the right to the conscience clause differs from the freedom of conscience. While the former is a legal norm that permits the individuals to refuse to perform a medical act that offends their moral beliefs [8], the latter is an ethical norm and a fundamental value which results directly from human dignity [9].

While in the internal realm of thought (*forum internum*) freedom of conscience and religion remains a matter of individual choice and belief, subject to no restrictions, in the external realm (*forum externum*) one’s right to manifest these beliefs may be subject to legal restrictions, but only when “public security or order, public health and morality or the freedom and rights of other people are at risk” [6]. Indeed, while there may be good religious, moral, legal reasons to accommodate CC in the context of healthcare practice, it is often argued that the exercise of rights and beliefs of the HCPs may influence patients’ health or access to care services. It is also often claimed that as a result of CC the line between the public and the private space becomes blurred [10, 11].

CC has been included in the provisions of Polish medical law since 1996 and is regulated by art. 39 of the *Act on the Professions of Doctors and Dentists*, art. 12 of *the Act on the Professions of Nurse and Midwife* and the *Code of Medical Ethics* [12–15]. The scope of its application, however, is narrow, as it applies only to selected groups of healthcare professionals: physicians, nurses and midwives.

Although the conditions for using CC are included in the provisions of Polish medical law, this topic continues to be a contentious subject. In fact, the problem comes to the fore every time abortion, contraception and IVF are discussed. It gained momentum, however, in 2014 when nearly four thousand Polish doctors and medical students signed the Declaration of faith of Catholic doctors and students of medicine *on the* sexuality and fertility of human beings, in which they affirm the Catholic Church’s teaching that all human life is sacred from the moment of conception and declare their refusal to provide abortions, contraception or *in vitro* fertilization on religious grounds [16, 17].

Unsurprisingly, the document provoked a heated debate and great concerns among Polish society, and, according to the *Center for Public Opinion Research*, the majority of Poles (73%) believed that even in the case of a conflict of conscience, the physician should not refuse to refer to prenatal tests when there is an increased risk of a genetic or developmental defect in a fetus. Similarly, 62% of respondents believed that a physician should not refuse a referral for abortion if it is not restricted by the law. According to 55% of respondents a physician should not refuse to prescribe contraceptives and 52% believed that he or she should not refuse to perform an abortion when it is legal [18].

Although previous research focused on the opinions of the physicians and medical students much less is known about the attitudes of nurses and pharmacists regarding the conscience clause. While physicians coordinate patient care, other HCPs, including nurses, midwifes and pharmacists face moral dilemmas related to patient care [18–25].

The objective of the study was therefore to examine the association between religion and physicians’, nurses’ and pharmacists’ attitudes towards conscience clauses in Poland. The rationale for choosing these groups rests on the fact that, while in current Polish law CC applies to physicians and nurses, there is an ongoing debate whether pharmacists should also have the right to refuse to dispense drugs related to contraception, and the “morning-after pill” in particular.

At the same time, it should be noted that at the time of this research abortion was legal in Poland on three conditions: when pregnancy poses a threat to the life or health of the pregnant woman, when prenatal tests or other medical indications suggest a high probability of a severe and irreversible impairment in the fetus or an incurable life-threatening disease and when there is a justified suspicion that the pregnancy resulted from a prohibited act, i.e., rape [26]. After the judgment of the Polish Constitutional Tribunal in October 2020 the second premise for allowing legal abortion has been declared unconstitutional and is no longer allowed [27].

According to Polish medical law, a physician is obliged to provide immediate medical assistance to a patient, especially when a failure to do so would pose a threat to the patient’s health or life [12]. The first justification for legal abortion, i.e., when the pregnancy threatens the mother’s life or health, may be considered a compulsory service, which causes no HCPs to invoke CC. There are, however, differences among Polish medical lawyers on this subject [28–30]. The other cases mentioned in the act raise similar doubts. Since there is no clear decision as to whether the above-mentioned legal cases should be treated as mandatory or permitted benefits, we have decided to investigate the opinion of HCPs on this subject.

## Methods

### Study Design

This research included data from an anonymous, self-administered questionnaire regarding HCPs’ attitudes towards the conscience clause. While it examines the association between religion and HCPs’ attitudes towards CC it was designed as a pilot survey aimed to test questionnaire and see they would respond to the survey design and whether our questions will generate useful results.

### Participants and Setting

The study was conducted between January and March 2020 among practicing physicians, nurses and pharmacists in Poznan, Poland. Respondents were recruited during specialization courses or training sessions organized by the Poznan District Chamber of Nurses and Midwives, the Wielkopolska Regional Chamber of Pharmacy and the Department of Gynecological Oncology, Poznan University Hospital of Lord’s Transfiguration. After receiving the final approval an online survey was distributed to physicians, nurses and pharmacists taking their specialization courses and trainings. Each of these courses consisted of 100 participants who agreed to took part in the survey.

### Research Tools

A standard questionnaire was used, comprising topics based on literature reviews and the objective of the study. The survey questionnaire consisted of three parts. The first part dealt with the demographic characteristics of the study participants. The second part gathered information on the respondents’ opinions regarding the legal aspects of the CC. We asked whether the current law precisely defines a set of rules for HCPs who use the conscience clause, the subject areas of which are not fully regulated, which HCPs should be able to use the CC and for excludes respondents using the CC in cases of legal abortion. The third part of the questionnaire concerned the HCPs’ personal experiences with CC. We therefore asked whether respondents had ever invoked the CC or whether they intended to do so. Respondents were also asked whether they have ever found themselves in a situation that required them to perform professional activities inconsistent with their conscience, and whether employers should have the right to ask about the moral beliefs of the candidates applying for jobs.

Religiosity was measured with one item: the role of religion in personal life. Based on their responses, HCPs were divided into two groups: religious (HCPs for whom religion was important in their life decisions and choices) and ambivalent/nonreligious (HCPs who either declared separation of their religious beliefs from their life decisions and choices or who felt religion was irrelevant to them).

### Data Collection

The final version of the questionnaire was distributed among HCPs in several healthcare facilities in Poznan, Poland. All HCPs, received an invitation letter and were informed about the study’s purpose, as well as the voluntary, anonymous, and confidential character of the study. Participants completed self-administered paper-pencil survey. Questionnaires took approximately 20 min to complete and were collected anonymously.

### Ethical Issues

The study was performed in line with the principles of the Declaration of Helsinki. Although in accordance with local legislation and national guidelines on research involving human subjects, ethical approval was not required it was obtained from the Department of Gynecological Oncology Poznan University Hospital of Lord’s Transfiguration (LBK/63/2018), from the Wielkopolska Regional Chamber of Pharmacy in Poznan (Wlkp. OIA/2711/2018), and from Poznan District Chamber of Nurses and Midwives (OIPIP 15/3/2019). Additionally, ethics approval and research governance approval were obtained from the Poznan University of Medical Sciences Bioethics Committee. Informed consent was also obtained from all respondents enrolled in the study.

### Data Analysis

The data collected in the questionnaires were verified and checked for completeness, quality and consistency, and exported into the statistical packages JASP (Version 0.16.1.0) and Dag-stat. The results were presented as descriptive statistics. Tables 2, 6 display the number of responses to individual questions along with the corresponding percentages of the total. A Likelihood Ratio Chi-square was used to assess the differences in the distribution of answers among the groups. A 5% level of significance was used for all the hypothesis tests.

## Results

The study group consisted of representatives of three healthcare professionals groups, 100 each of physicians, nurses and pharmacists (Table 1). The majority of respondents were women (81%) with a mean seniority of 10.75 years. Most of our respondents (89.7%) hold a university degree, as, since 2004, the Bologna Process has determined that all HCPs must be university graduates, so only those who began work earlier, e.g., as nurses, may have other qualifications. More than half of respondents (54%) were living in cities of over 500,000 inhabitants. Although the largest group of respondents declared themselves practicing believers (43.7%) only 14.3% declared that religion played a significant role in their life.

**TABLE 1:** Participant demographic characteristics (Poland, 2020).

Characteristics	*N* (%)
Number of respondents	300
Sex	
Female	243 (81)
Male	57 (19)
Mean age	35.34
Minimum age	22
Maximum age	61
SD	9.26
Job seniority	
Mean seniority	10.75
Minimum seniority	0.17
Maximum seniority	38
SD	9.52
Education	
University	269 (89.7)
High school	31 (10.3)
Domicile	
Up to 10,000 inhabitants	43 (14.4)
10–50,000 inhabitants	26 (8.7)
51–100,000 inhabitants	25 (8.3)
101–500,000 inhabitants	44 (14.7)
Above 500,000 inhabitants	162 (54)
Religious practices	
Believing/practicing	131 (43.7)
Believing/not practicing	99 (33)
Non-believer/practicing	10 (3.3)
Non-believer/not practicing	60 (20)
What role does religion play in your life?	
Significant, it influences my life decisions and choices	43 (14.3)
Rather big, I try to follow religious principles in my life	85 (28.3)
Little, I separate religion from public issues	115 (38.3)
None, it is irrelevant to me	57 (19)

At the same time based on HCPs’ declarations regarding their religious beliefs and the role of religion in their life, the respondents were divided into two groups: religious (HCPs who declared that religion influences their life decisions and choices) versus ambivalent/non-religious (HCPs who had no religious allegiences or felt religion was irrelevant to them). The comparison between these two groups showed that they did not differ in terms of sex; however, respondents who declared that religion played an important role in their life tended to come from smaller towns and villages and were older. Nurses were also overrepresented.

The majority of HCPs in both groups believed that the current Polish law is too imprecise in its definition of the rules that guide their use of the conscience clause (57.8% and 60.5%, respectively) (Table 2). Religious HCPs in particular, however, complained that it remains rather unclear what groups of HCPs have the right to invoke the conscience clause. They also stressed that the conditions necessary for using it should be more precise.

**TABLE 2:** Respondents’ opinions concerning legal aspects of the CC (Poland, 2020).

	Religious HCPs		Ambivalent/non-religious HCPs		*p*
	*N*	%	*N*	%	
Do you think that the current law describes a precise set of rules established for healthcare professionals who might seek to use the CC?					ns
Yes	12	9.4	16	9.3	
No	74	57.8	104	60.5	
I have no opinion	42	32.8	52	30.2	
If not, what remains to be fully regulated?					
Determining the scope, i.e., determining which group of medical professionals has the right to invoke the CC	57	77	65	62.5	**<0.05**
Establishing the conditions that must always be met if a medical professional seeks to invoke the CC	67	90.5	83	79.8	**<0.05**
Measures to ensure that the patient has practical access to a specific health service	49	66.2	81	77.9	ns
All of the above-mentioned circumstances	41	55.4	54	51.9	ns
Other	2	2.7	1	1	ns
In your opinion, what issues are pertinent to the CC?					
Abortion	124	96.9	137	79.6	**<0.001**
Prenatal testing	44	34.4	45	26.2	ns
Contraception	73	57	75	43.6	**<0.05**
Assisted reproduction techniques	65	50.8	62	36	**<0.01**
Transplantology	40	31.2	40	23.2	ns
Palliative care	51	39.8	49	28.5	**<0.05**
Other	3	2.3	4	2.3	ns
None of the above	3	2.3	35	20.3	**<0.001**

Statistically significant differences are written in boldface; ns, not significant.

At the same time, HCPs who defined themselves as religious argued that the provision of the conscience clause should mostly be enacted in connection with issues relating to abortion, reproduction, contraception, assisted reproduction techniques and palliative care. Non-religious HCPs, however, frequently declared that none of issues mentioned in the survey should be covered by the CC (20.3% and 2.3%, respectively).

Both groups differed in their opinion regarding which professional groups should be covered by the conscience clause (Table 3). Religious respondents indicated physicians (88.3% vs. 44.2%) and pharmacists (33.3% vs. 17.9%) more often. In reference to nurses no statistically significant difference was found (87.1% vs. 80.6%).

**TABLE 3:** Respondents’ opinion to whom the CC should apply (Poland, 2020).

	Religious HCPs		Ambivalent/non-religious HCPs		*p*
	*N*	%	*N*	%	
Physicians					**<0.001**
Yes	113	88.3	76	44.2	
No	15	11.7	96	55.8	
Nurses					ns
Yes	81	87.1	54	80.6	
No	12	12.9	13	19.4	
Pharmacists					**<0.05**
Yes	31	33.3	12	17.9	
No	62	66.7	55	82.1	

Statistically significant differences are written in boldface; ns, not significant.

When asked about their personal experience with the CC, it was religious HCPs who declared having exercised the conscience clause more often (10.2% vs. 2.9%). They also intended to do so more frequently (20.6% vs. 7.8%) (Table 4). Religious HCPs were also more concerned about the personal consequences of using this right both in terms of losing their job or being passed over for promotion and deterioration of relations with coworkers. Finally, more religious respondents declared having been in a situation that required them to act against their conscience (23.4% vs. 12.8%)

**TABLE 4:** HCPs’ personal experiences with the CC (Poland, 2020).

	Religious HCPs		Ambivalent/non-religious HCPs		*p*
	*N*	%	*N*	%	
Respondents who have invoked the CC					<0.01
Yes	13	10.2	5	2.9	
No	115	89.8	167	97.1	
Respondents who intended to invoked the CC					<0.001
Yes	26	20.6	13	7.8	
No	89	77.4	154	92.2	
If there were concerns, what were they?					
I may lose my job	21	16.4	8	4.6	<0.001
I cannot get promotion	6	4.7	1	0.6	<0.05
Deterioration of personal relations with my coworkers	19	14.8	9	5.2	<0.01
I will lose respect	10	7.8	7	4.1	ns
Other	5	3.9	0	0	
Have you ever been in a situation that required you to perform a medical service that was against your conscience?					<0.05
Yes	30	23.4	22	12.8	
No	98	76.6	150	87.2	
Do you think that the employer should be entitled to ask an applicant about his or her private beliefs about the CC during a job interview?					<0.001
Yes	36	28.1	80	46.5	
No	78	60.9	69	40.1	
I have no opinion	14	11	23	13.4	
Should the law oblige healthcare professionals who have refused to perform a medical procedure on ethical grounds to refer the patient to another specialist?					<0.001
Yes	59	46.1	120	69.8	
No	54	42.2	28	16.3	
I have no opinion	15	11.7	24	13.9	

Statistically significant differences are written in boldface; ns, not significant.

On the other hand, ambivalent/nonreligious HCPs agreed that the employer should have the right to ask an applicant about his or her private beliefs about the conscience clause during a job interview (46.5% vs. 28.1%), and that the law should oblige those HCPs who have refrained from performing a medical procedure due to ethical objections to refer a patient to another specialist (69.8% vs. 46.1%).

Some significant differences were also found in relation to the reasons both groups felt were legitimate for performing obligatory abortions (Table 5). Ambivalent/non-religious HCPs supported abortion in all three legal cases permitted by Polish law more often. They also supported the procedure for all eventualities.

**TABLE 5:** Cases in which in the respondents’ opinion HCPs should be unable to invoke the CC (Poland, 2020).

	Religious HCPs		Ambivalent/non-religious HCPs		*p*
	*N*	%	*N*	%	
Pregnancy constitutes a threat to the mother’s life or represents a serious threat to her health					
Molar pregnancy	52	52	74	75.5	<0.001
Trophoblast tumors	52	52.5	79	80.6	<0.001
Cervical cancer	42	42	71	72.4	<0.001
Placenta previa	30	30	57	58.2	<0.001
Placental abruption	30	30.6	61	62.2	<0.001
Bleeding from varicose veins (vagina and vulva)	21	21.2	35	36.1	<0.05
Velamentous cord insertion	29	29	48	49.5	<0.01
Ectopic pregnancy	64	63.4	86	86.9	<0.001
Fatal or severe fetal impairment					
Down syndrome	11	10.9	36	36.7	<0.001
Hemophilia	6	6	26	26.8	<0.001
Silver-Russell syndrome	13	12.8	39	39.8	<0.001
Klinefelter’s syndrome	9	8.9	39	39.8	<0.001
Huntington’s disease	8	7.9	37	37.7	<0.001
Edwards syndrome	19	18.8	53	54.1	<0.001
Angelman syndrome	11	10.9	42	43.7	<0.001
Marfan syndrome	8	7.9	40	41.2	<0.001
Patau’s syndrome	22	21.8	53	54.6	<0.001
Turner syndrome	7	6.9	33	33.7	<0.001
Color-blindness	2	2.1	21	21.6	<0.001
Other genetic diseases	2	4.2	8	20	<0.05
Isolated defects of one system or organ	4	4.1	28	29.2	<0.001
Spina bifida or non-corrective heart defects	15	15	56	56.6	<0.001
Social and economic reasons					
Rape	43	43	73	73.7	<0.001
Incest	36	36	72	72.7	<0.001
Pedophilia	45	44.5	73	73.7	<0.001
Difficult living conditions	3	3	23	23.5	<0.001
Difficult personal situation	3	3	23	23.5	<0.001

Statistically significant differences are written in boldface; ns, not significant.

In the case of the CC regarding pharmacists, significant differences have been observed (Table 6). While religious HCPs argued that pharmacists should be granted the right to refuse to dispense drugs related to contraception on moral or religious grounds more often (64.7% vs. 38.1%), ambivalent/nonreligious respondents declared that a pharmacist’s refusal to fill a prescription for birth control would incur their objection (83.7% vs. 60.9%). At the same time, the former indicated an intrauterine device (IUD) (29% vs. 12.7%) and the “morning-after pill” (49.3% vs. 24.5%).

**TABLE 6:** Respondents’ opinions regarding the CC for pharmacists (Poland, 2020).

	Religious HCPs		Ambivalent/non-religious HCPs		*p*
	*N*	%	*N*	%	
For which drugs would the pharmacist be entitled to invoke the CC?					
Hormonal contraceptive drugs	33	64.7	8	38.1	**<0.05**
Sleeping pills and sedatives	8	15.7	4	14.3	ns
Antidepressants	2	3.9	5	23.8	**<0.01**
Anxiolytic drugs	2	3.9	0	0	ns
Painkillers	2	3.9	2	9.5	ns
Anesthetic drugs	3	5.9	3	14.3	ns
Drugs that stimulate the central nervous system	11	21.6	7	33.3	ns
Other drugs	7	13.7	3	14.3	ns
For all drugs, regardless of their profile	9	17.6	4	19	ns
Would a pharmacist’s failure to fill a prescription for contraceptives available in pharmacies raise your objection?					**<0.001**
Yes	78	60.9	144	83.7	
No	38	29.7	23	13.4	
I have no opinion	12	9.4	5	2.9	
Refusal to fill the following contraceptives would rise my objection					
Hormonal contraceptives	4	5.8	1	0.9	ns
Mechanical methods of contraception, e.g., vaginal rings, condoms	9	13	6	5.4	ns
Chemical barrier methods, e.g., globules, foams, creams, spermicidal fluids	8	11.6	6	5.4	ns
Methods that prevent fertilization, e.g., IUD	20	29	14	12.7	**<0.01**
Postcoital contraception (the “morning-after pill”)	34	49.3	27	24.5	**<0.001**

Statistically significant differences are written in boldface; ns, not significant.

## Discussion

Although over the last two decades, there has been a shift in religiosity among Poles, Poland remains an overwhelmingly religious Roman Catholic nation. Indeed, even though the percentage of those who practice regularly has decreased to 43%, still 91% of Poles declare themselves Catholics [31, 32]. *Simultaneously*, *due to secularization and the privatization of religion many Poles* are moving towards a more secular view of life, one with a greater separation between the Church and the state, and a rejection of Church mandates on individual morality. Consequently, although religion remains at the core of the value system for many Poles and a source of cultural identification, a “*secularization of morality*” *is to be observed*, as the increasing number of Poles feel no need to justify the moral rules of conduct with reference to religion and espouse moral opinions in sharp contrast with their religion [33]. A growing trend towards moral relativism and “situational morality,” may be noticed, as many Poles argue that in some situations moral rules are suspended, that decisions about good and evil are a personal matter and depend on particular circumstances [34]. However, this should not surprise since similar situation was observed in other countries where churches influenced discussion and legislation on such issues as (sexual) education, abortion, divorce, stem cell research, or same-sex marriage, often despite mass social protests [35, 36].

At the same time studies suggest that religiosity still influences HCPs’ attitudes towards patients. For example, Pawlikowski et al. showed that religious faith influences physicians’ professional decisions and is an important coping resource in difficult professional situations. While religious ideas may inspire some to become physicians, religiosity correlates positively with altruism, empathy and holistic encounters with patients, they may clash with respect for patients’ autonomy [37].

The importance of religiosity in making therapeutic decisions and empathetic approaches was also found among nurses [38]. Wenger and Carmel also demonstrated that physicians’ religiosity might affect their end-of-life views which, in turn, directly translate into attitudes towards end-of-life care and influence the way their patients die. Very religious physicians were more likely to continue life-sustaining treatment and were also less eager to withdraw care from their patients. They were also less likely to approve prescribing necessary analgesia if it might hasten patients’ death [39]. Both Balslev van Randwijk et al. and Emanuel et al. found that religiosity and spirituality determine physicians’ attitudes toward such end-of-life procedures as euthanasia, physician-assisted suicide, sedation into unconsciousness in dying patients and withdrawal of life support [40, 41]. These conclusions were also confirmed by research from Canada. Brown et al. believe that faith and spiritual beliefs are an important factor influencing the lack of participation of the surveyed physicians and nurses in formal medical assistance in dying processes. Some HCPs have advocated aligning their clinical practice with faith or spiritual beliefs because they provide a source of moral responsibility as well as inner strength and comfort with dying and death [42].

Other research suggest that religiosity is a powerful predictor of HCPs’ willingness to perform abortions [43]. Davidson et al. found that pharmacists’ religiousness affected their willingness to dispense contraceptives and abortion medications [44]. Davis et al. showed that nurses who declared their ethical beliefs to be founded on their religion experienced higher levels of moral anxiety than nurses whose beliefs were shaped by more secular factors, such as family values, political views or a professional code of ethics [45]. Finally, a study by Domaradzki and Walkowiak showed that medical students’ religiosity played a key role in determining their motivations to join the fight against the COVID-19 pandemic, as those who declared themselves religious tended to be inspired by altruistic motivations rather than by personal or selfish motives [46].

This research confirms findings from other European countries, such as Italy, where approximately 70% of HCPs including physicians, nurses and midwives, and medical students support the CC [25, 47]. For example, over 45% of medical students in the United Kingdom [20], 79.2% in Spain supported HCP’s right to CC [22], as did 89.0% of Slovakian pharmacists and pharmacy students [48]. A Polish study conducted by Baranowska et al. showed that 65% of pharmacy students and 50% of practicing pharmacists were in favour of introducing CC for pharmacists [49].

According to the participants of the study, CC is rarely utilized in Poland. Respondents also declared that they find it extremely hard to use CC in the context of healthcare practice. While in Slovakia, too, only 32.5% of pharmacists declared having implemented CC in practice [48], studies conducted by Lamb et al. also showed that, although Canadian nurses in Ontario expected the right to CC, they complained over lack of support, especially in situations related to conflicting viewpoints with colleagues and professionally sanctioned practices, such as medical assistance in dying [50, 51]. Many HCPs enrolled in this study would nevertheless be willing to use their right to CC. Piecuch et al. similarly showed that, although 92% of Polish pharmacists have never refused to fill a prescription due to their beliefs, 15% would exercise this right if it were legal [52].

Another important finding is that in accord with previous studies this research shows that among the reasons for accepting HCPs’ right to CC were carrying out or assisting in (early) abortions, euthanasia and assisted reproduction [48, 53]. At the same time, according to our respondents, contraception came second to abortion as a reason HCPs should be entitled to invoke CC. This right was also recognized by both religious and non-religious respondents (57% and 43.6%, respectively). Post-coital contraception or the “morning-after pill” and intrauterine contraceptive devices (ICD) were therefore indicated as products pharmacists should have the right to refuse to sell on the basis of their conscience. This supports findings from a previous study conducted by Piecuch et al. who demonstrated that, according to Polish pharmacists, medical products that should be subjected to CC were primarily emergency contraception (65%), vaccines (46%), intrauterine devices (27%) and hormonal contraception (21%). Similar results were also found in other European countries, such as England, Italy, Portugal and Norway which have similar provisions regarding the legal regulation of CC [54].

On the other hand, this research shows that many HCPs in Poland still believe that HCPs should not be allowed to refuse to perform a medical service which is permitted by the law, but which offends their conscience or personal beliefs. For example, although the majority of Norwegian medical students would object to participating in euthanasia (89%), abortion (55%) or ritual circumcision for boys (52%), many believed that physicians should not be allowed to refuse to participate in such procedures on the grounds of conscience and their general tolerance towards CC was low [21]. Similarly, 70.8% of Spanish students would not invoke CC in cases of abortion; 70% believed that it might have harmful consequences for pregnant women [22]. According to Shaw and Downie, nurses who declare themselves to be religious tend to consider their right to CC more important than the patients’ healthcare rights [55].

Respondents therefore seemed to be aware of their dual commitment toward their conscience and HCPs’ obligations towards patients’ health and rights. For that reason many declared that HCPs must somehow safeguard both HCPs’ right to CC and patients’ (reproductive) rights and health [10]. Piecuch et al. also found that, while 73% of Polish pharmacists believed that pharmacists should not have the right to CC, respondents who supported the implementation of the CC would grant this right only if the pharmacists were obliged to present other real options to the patient about obtaining a specific product. Similarly, the majority of Polish society argues that in the case of a conflict of conscience the physician cannot refuse to prescribe contraceptives or perform an abortion when it is legal [18].

While in some European countries, i.e., United Kingdom, an additional system for monitoring healthcare services has been implemented, respondents from this study claimed that during the job interview employers should not have the right to ask HCPs about their personal beliefs and medical services or pharmaceutical products to which they may have ethical objections [56–59]. They were also concerned that refusal to perform some procedures might cost them their jobs and provoke conflicts with other HCPs.

Most importantly, this research shows that religion has the greatest influence on shaping HCPs’ attitudes towards CC. For example, studies conducted among pharmacists in the United Kingdom by Cooper et al. and Maxwell et al. revealed that religion might affect their ethical attitudes towards selling emergency hormonal contraception [60, 61]. A study by Toro-Flores et al. conducted among Spanish nurses also demonstrated that nurses who declared themselves believers supported CC much more often, in thirteen out of eighteen possible cases, including abortion within the first 14 weeks of pregnancy, voluntary sterilization, the dispensing of the morning-after pill, voluntary abortions in cases when the pregnancy occurred due to rape or when there is the assumption of fetus malformation or when there is a health risk to the mother [53]. A recent study among Greek nurses by Voultsos et al. also showed that religion, along with the quality of education received and upbringing, were the factors that had the greatest impact on the choice of values shaping the morality of the respondents, also in the field of conscientious objection. However, raising ethical objections in the workplace is related to the experience of lack of support and suboptimal communication, which increase fears of bullying and negative gossip, creating a barrier to exercising the principle of conscience clause [62].

At the same time, Bouthillier and Opatrny showed that physicians’ refusal to participate in medical aided dying was not based on moral or religious grounds, but rather resulted from the emotional burden related to this act and the fear of psychological repercussions [23]. Strickland also demonstrated that while 45.2% of future doctors believed that physicians should be entitled to conscientiously object to any procedure for which they have a moral, cultural or religious disagreement, the majority of the objections were for non-religious reasons [20]. Finally, a systematic review conducted by Fleming et al. indicated that the main reasons for or against CC to abortion provision expressed by nurses or midwives were moral reasons which exceeded practical, religious and legal reasons [63, 64].

Although this research showed that of twenty seven possible reasons for abortion there were significant difference in all of them, and that religion was the main predictor for those differences, it should also be noted that neither religious nor non-religious respondents accepted or rejected all the indications. Just as religious HCPs did not close their minds to mandatory abortion for all reasons, so non-religious respondents were unwilling to accept the procedure in all situations approved be the law.

This study therefore shows, that HCPs’ attitudes towards CC is much more complex and cannot be explained solely on the basis of the respondents’ attitudes towards religion. A detailed analysis of their answers indeed suggests that respondents opinions on the admissibility of abortion depends on particular circumstances and reflect what was said about situational morality. HCPs’ acceptance or rejection of abortion is some situations, i.e., ectopic pregnancy, certainly does not automatically entail their acceptance or rejection of other situations, i.e., cervical cancer. This supports findings from a previous study, which demonstrated that, while many Polish gynecologists accepted performing abortions due to lethal defects (46%) or serious diseases in the mother (34%), only 21.1% referred to the religious aspect of abortion and 6% claimed that the procedure offended their conscience [24].

### Study Limitations

Although to the best of our knowledge this is one of the few studies on the attitudes of Polish HCPs towards CC, it has limitations which may have an impact on their generalizability and interpretation. Firstly, although this survey was designed as a preliminary study, still only 300 HCPs, which may have an impact on whether the results might be extrapolated and interpreted. Consequently, while this study represents solely the opinions of those HCPs who agreed to participate in the study it would be desirable to compare the findings with those from a survey conducted on a larger sample size. Secondly, HCPs from only one Polish city were enrolled in the study. The study therefore has a local dimension. Thus, it would be desirable to compare the findings from other parts of the country. Thirdly, because the COVID-19 pandemic hindered the recruitment process and reduced the number of respondents who took part in the specialization courses or training sessions the results cannot therefore be extrapolated for the entire population of HCPs either in Poznan or in Poland as a whole. Consequently, it would be desirable to compare the findings from larger group. Fourthly, since this study rests on the quantitative method only, to understand better the association between religion and healthcare practitioners’ attitudes towards conscience clauses further in-depth studies using qualitative methods would be required. Finally, because respondents were not asked specific questions regarding their religious beliefs, their religiosity was assessed on their declarations alone. Thus, future studies should also use well-validated instruments to measure religiosity.

Despite these limitations, however, this study also enjoys some advantages. Most importantly, as there is a scarcity of previous work on the topic, this research helps fill the gap in the research on the attitudes of Polish HCPs towards CC and it may stimulate further research on the topic.

### Conclusion

While this study shows that HCPs’ religiosity was a significant predictor of acceptance of CC, it also influenced their opinions on healthcare professions who they feel should hold the right to CC and medical services that might be denied on moral grounds. None of these groups, however, was homogeneous, as, while some religious HCPs believed that some medical services, i.e., contraception, assisted reproduction techniques or palliative care should be excluded from those that might permit HCPs from invoking CC, some non-religious HCPs supported the right to conscious objection. Religious HCPs also admitted to having used CC more often and being willing to do so in a situation of moral conflict. Finally, they were more concerned over the personal consequences of using the right to CC in a medical setting. At the same time, both religious and non-religious HCPs believed that the Polish regulations regarding CC are unclear and inaccurate.

## Data Availability

The datasets generated and analyzed during the current study are available from the corresponding author on reasonable request.

## References

[1] United Nations. Universal Declaration of Human Rights (1948). Available From: https://www.un.org/sites/un2.un.org/files/udhr.pdf (Accessed August 9, 2023).

[2] Council of Europe. European Convention on Human Rights (1953). Available From: https://www.echr.coe.int/documents/convention_eng.pdf (Accessed August 9, 2023).

[3] United Nations. International Covenant on Civil and Political Rights (1976). Available From: https://www.ohchr.org/sites/default/files/ccpr.pdf (Accessed August 9, 2023). 12289227

[4] Safjan M. Wolność Religijna W Konstytucjach Państw Europejskich. In: Krukowski J Theisen O, editors. Kultura I Prawo: Materiały III Międzynarodowej Konferencji Na Temat „Religia I Wolność Religijna W Unii Europejskiej” (2003). Warszawa: Towarzystwo Naukowe Katolickiego Uniwersytetu Lubelskiego (2022).

[5] Waszczuk-Napiórkowska J. Opinia Prawna Dotycząca Wolności Sumienia W Konstytucjach Krajów Unii Europejskiej. Zeszyty Prawnicze (2012) 3(35):231–3.

[6] KONSTYTUCJA. Konstytucja Rzeczypospolitej Polskiej (1997). Available From: https://www.sejm.gov.pl/prawo/konst/polski/kon1.htm (Accessed August 9, 2023).

[7] Galewicz W. Jak Rozumieć Medyczną Klauzulę Sumienia? Diametros (2012) 34:136–53. 10.13153/diam.34.2012.503

[8] Głusiec W. Klauzula Sumienia dla Farmaceutów: Analgia Opinii Wydanej Przez Comitato Nazionale pre la Bioethica. Diametros (2012) 32:62–76. 10.13153/diam.32.2012.477

[9] Orzeszyna K. Klauzula Sumienia Jako Gwarancja Realizacji Prawa Do Wolności Sumienia. Medyczna Wokanda (2017) 9:17–29.

[10] Chavkin W Leitman L Polin K. Global Doctors for Choice. Conscientious Objection and Refusal to Provide Reproductive Healthcare: A White Paper Examining Prevalence, Health Consequences, and Policy Responses. Int J Gynecol Obstet (2013) 123(3):41–56. 10.1016/S0020-7292(13)60002-8 24332234

[11] Shanawani H. The Challenges of Conscientious Objection in Health Care. J Religion Health (2016) 55(2):384–93. 10.1007/s10943-016-0200-4 26923838

[12] USTAWA. Ustawa O Zawodach Lekarza I Lekarza Dentysty (1996). Available From: https://isap.sejm.gov.pl/isap.nsf/download.xsp/WDU19970280152/U/D19970152Lj.pdf (Accessed August 9, 2023).

[13] USTAWA. Ustawa O Zawodach Pielęgniarki I Położnej (2011). Available From: https://isap.sejm.gov.pl/isap.nsf/download.xsp/WDU20111741039/U/D20111039Lj.pdf (Accessed August 9, 2023).

[14] Kodeks etyki lekarskiej. Kodeks Etyki Lekarskiej (2004). Available From: https://nil.org.pl/uploaded_images/1574857770_kodeks-etyki-lekarskiej.pdf (Accessed August 9, 2023).

[15] Czekajewska J. Ethical Aspects of the Conscience Clause in Polish Medical Law. Kultura i Edukacja (2018) 4(122):206–20. 10.15804/kie.2018.04.13

[16] Półtawska W. Declaration of Faith of Catholic Doctors and Students of Medicine, on the Sexuality and Fertility of Human Beings. Linacre Q (2015) 82(2):99–100. 10.1179/0024363914Z.000000000111 25999605 PMC4434787

[17] Olszówka M. Analgia Projektu Ustawy O Zmianie Ustawy O Zawodach Lekarza I Lekarza Dentysty, Ustawy O Diagnostyce Laboratoryjnej Oraz Ustawy O Zawodach Pielęgniarki I Położnej, Zawartego W Druku Senackim Nr 1034/IX Kadencja (Sprzeciw Sumienia). Studia z Prawa Wyznaniowego (2019) 22:349–77. 10.31743/spw.4788

[18] Centrum Badania Opinii Społecznej. Klauzula Sumienia Lekarza I Farmaceuty (2014). Available From: https://www.cbos.pl/SPISKOM.POL/2014/K_094_14.PDF (Accessed August 9, 2023).

[19] Pellegrino ED. The Physician's Conscience, Conscience Clauses, and Religious Belief: A Catholic Perspective. Fordham Urban L J (2002) 30(1):221–44. 15868671

[20] Strickland SL. Conscientious Objection in Medical Students: A Questionnaire Survey. J Med Ethics (2012) 38(1):22–5. 10.1136/jme.2011.042770 21768618

[21] Nordstrand SJ Nordstrand MA Nortvedt P Magelssen M. Medical Students’ Attitudes Towards Conscientious Objection: A Survey. J Med Ethics (2014) 40(9):609–12. 10.1136/medethics-2013-101482 23946469

[22] Soengas AM Ramos AC. Percepción del Alumnado de Medicina Sobre la Objeción de Conciencia a la Interrupción Voluntaria del Embarazo en Galicia. Gaceta Sanitaria (2019) 34(2):150–6. 10.1016/j.gaceta.2019.02.007 30962031

[23] Bouthillier ME Opatrny L. A Qualitative Study of Physicians’ Conscientious Objections to Medical Aid in Dying. Palliat Med (2019) 33(9):1212–20. 10.1177/0269216319861921 31280666

[24] Zaręba K La Rosa VL Kołb-Sielecka E Ciebiera M Ragusa R Gierus J Attitudes and Opinions of Young Gynecologists on Pregnancy Termination: Results of a Cross-Sectional Survey in Poland. Int J Environ Res Public Health (2020) 17(11):3895. 10.3390/ijerph17113895 32486362 PMC7311986

[25] Autorino T Mattioli F Mencarini L. The Impact of Gynecologists’ Conscientious Objection on Abortion Access. Soc Sci Res (2020) 87:102403. 10.1016/j.ssresearch.2020.102403 32279862

[26] USTAWA. Ustawa O Planowaniu Rodziny, Ochronie Płodu Ludzkiego I Warunkach Dopuszczalności Przerywania Ciąży (1993). Available From: https://isap.sejm.gov.pl/isap.nsf/download.xsp/WDU19930170078/U/D19930078Lj.pdf (Accessed August 9, 2023).

[27] Makowska M Boguszewski R Sacharczuk K. A Study of Opinions About the Polish Constitutional Tribunal’s Judgement Strengthening Polish Abortion Laws. Eur J Contraception Reprod Health Care (2021) 27(1):39–44. 10.1080/13625187.2021.1980872 34590965

[28] Boratyńska M. O Łamaniu Przepisów Dopuszczających Przerywanie Ciąży. Prawo i Medycyna (2008) 10(2):88–103.

[29] Nawrot O. Prawa Człowieka, Sprzeciw Sumienia I Państwo Prawa. In: Stanisz P Pawlikowski J Ordon M, editors. Sprzeciw Sumienia W Praktyce Medycznej – Aspekty Etyczne I Prawne. Lublin: KUL (2014). p. 105–16.

[30] Bosek L. Problem Zakresowej Niekonstytucjonalności Art. 39 Ustawy O Zawodach Lekarza I Lekarza Dentysty. In: Stanisz P Pawlikowski J Ordon M, editors. Sprzeciw Sumienia W Praktyce Medycznej – Aspekty Etyczne I Prawne. Lublin: KUL (2014). p. 87–104.

[31] Centrum Badania Opinii Społecznej. Religijność Polaków W Ostatnich 20 Latach (2020). Available From: https://cbos.pl/SPISKOM.POL/2020/K_063_20.PDF (Accessed August 9, 2023).

[32] Centrum Badania Opinii Społecznej. Źródła Zasad Moralnych (2022). Available From: https://www.cbos.pl/SPISKOM.POL/2022/K_001_22.PDF (Accessed August 9, 2023).

[33] Centrum Badania Opinii Społecznej. Zasady Moralne a Religia (2017). Available From: https://www.cbos.pl/SPISKOM.POL/2017/K_004_17.PDF (Accessed August 9, 2023).

[34] Centrum Badania Opinii Społecznej. Stosunek Polaków Do Wybranych Zjawisk I Zachowań Kontrowersyjnych Moralnie (2021). Available From: https://www.cbos.pl/SPISKOM.POL/2021/K_165_21.PDF (Accessed August 9, 2023).

[35] Grzymata-Busse A, Nations Under God: How Churches Use Moral Authority to Influence Policy. Princeton: Princeton University Press (2015). 10.2307/j.ctv7h0sfx

[36] Calkin S Kaminska ME. Persistence and Change in Morality Policy: The Role of the Catholic Church in the Politics of Abortion in Ireland and Poland. Feminist Rev (2020) 124:86–102. 10.1177/0141778919894451

[37] Pawlikowski J Sak JJ Marczewski K. Physicians’ Religiosity and Attitudes Towards Patients. Ann Agric Environ Med (2012) 19(3):503–7. 23020047

[38] Lankau A Krajewska-Kułak E Jankowiak B Baranowska A Bejda G. Effect of Nurses’ Religious Beliefs on Their Empathy and Life Satisfaction. Prog Health Sci (2017) 7(2):18–25. 10.5604/01.3001.0010.5717

[39] Wenger NS. Physicians' Religiosity and End-Of-Life Care Attitudes and Behaviors. Mount Sinai J Med (2004) 71(5):335–43. 15543435

[40] Balslev van Randwijk C Opsahl T Assing Hvidt E Bjerrum L Kørup AK Hvidt NC. Association Between Danish Physicians’ Religiosity and Spirituality and Their Attitudes Toward End-Of-Life Procedures. J Religion Health (2020) 59(5):2654–63. 10.1007/s10943-020-01026-3 32441014

[41] Emanuel EJ Fairclough D Clarridge BC Blum D Bruera E Penley WC Attitudes and Practices of U.S. Oncologists Regarding Euthanasia and Physician-Assisted Suicide. Ann Intern Med (2000) 133(7):527–32. 10.7326/0003-4819-133-7-200010030-00011 11015165

[42] Brown J Goodridge D Thorpe L Crizzle A. What Is Right for Me, Is Not Necessarily Right for You: The Endogenous Factors Influencing Nonparticipation in Medical Assistance in Dying. Qual Health Res (2021) 31(10):1786–800. 10.1177/10497323211008843 33938306 PMC8446887

[43] Awoonor-Williams JK Baffoe P Aboba M Ayivor P Nartey H Felker B Exploring Conscientious Objection to Abortion Among Health Providers in Ghana. Int Perspect Sex Reprod Health (2020) 46:51–9. 10.1363/46e8920 32375118

[44] Davidson L Pettis C Joiner A Cook D Klugman C. Religion and Conscientious Objection: A Survey of Pharmacists’ Willingness to Dispense Medications. Soc Sci Med (2010) 71(1):161–5. 10.1016/j.socscimed.2010.03.027 20447746

[45] Davis S Schrader V Belcheir M. Influencers of Ethical Beliefs and the Impact on Moral Distress and Conscientious Objection. Nurs Ethics (2012) 19(6):738–49. 10.1177/0969733011423409 22736753

[46] Domaradzki J Walkowiak D. Does Religion Influence the Motivations of Future Healthcare Professionals to Volunteer During the COVID-19 Pandemic in Poland? An Exploratory Study. J Religion Health (2021) 60:1507–20. 10.1007/s10943-021-01231-8 PMC8017435 33797729

[47] Kobyliński A. Aspekty Etyczno-Prawne Sporu O Klauzulę Sumienia We Włoszech W Latach 2014-2015. Studia Ecologiae et Bioethicae (2015) 13(3):25–48. 10.21697/seb.2015.13.3.02

[48] Ambrus T Kolář J Krempaská R. Výhrada Svědomí Při Výkonu Profese Lékárníka Ve Slovenské Republice. Ceska a Slovenska Farmaciei (2016) 65(5):176–81. 28073280

[49] Baranowska J Baranowski S Kuchta J Liwińska Z. Stanowisko Farmaceutów I Studentów Farmacji Wobec Klauzuli Sumienia. Pielęgniarstwo Polskie (2012) 4(46):187–9.

[50] Lamb C Babenko-Mould Y Evans M Wong CA Kirkwood KW. Conscientious Objection and Nurses: Results of an Interpretive Phenomenological Study. Nurs Ethics (2019) 26(5):1337–49. 10.1177/0969733018763996 29614913

[51] Lamb C Evans M Babenko-Mould Y Wong CA Kirkwood KW. Nurses' Use of Conscientious Objection and the Implications for Conscience. J Adv Nurs (2019) 75(3):594–602. 10.1111/jan.13869 30328136

[52] Piecuch A Gryka M Kozłowska-Wojciechowska M. Attitudes Towards Conscientious Objection Among Community Pharmacists in Poland. Int J Clin Pharm (2014) 36(2):310–5. 10.1007/s11096-013-9878-5 24234945 PMC3984665

[53] Toro-Flores R Bravo-Agüi P Catalán-Gómez MV González-Hernando M Guijarro-Cenisergue MJ Moreno-Vázquez M Opinions of Nurses Regarding Conscientious Objection. Nurs Ethics (2019) 26(4):1027–38. 10.1177/0969733017731915 29129123 PMC7323748

[54] Chavkin W Swerdlow L Fifield J. Regulation of Conscientious Objection to Abortion: An International Comparative Multiple-Case Study. Health Hum Rights (2017) 19(1):55–68. 28630541 PMC5473038

[55] Shaw J Downie J. Welcome to the Wild, Wild North: Conscientious Objection Policies Governing Canada's Medical, Nursing, Pharmacy, and Dental Professions. Bioethics (2014) 28(1):33–46. 10.1111/bioe.12057 24117541

[56] Balmer L. Royal Pharmaceutical Society and Conscientious Objectors. Lancet (2006) 367(9527):1980. 10.1016/S0140-6736(06)68884-3 16782487

[57] Deans Z. Conscientious Objections in Pharmacy Practice in Great Britain. Bioethics (2013) 27(1):48–57. 10.1111/j.1467-8519.2011.01918.x 21797914

[58] Merks P Szczęśniak K Świeczkowski D Blicharska E Paluch A Olszewska A Klauzula Sumienia Dla Farmaceutów W Środowisku Farmaceutów Praktykujących W Polsce I Wielkiej Brytanii. Farmacja Polska (2015) 71(8):2–9.

[59] Christopher C. Conscientious Objection and Healthcare in the UK: Why Tribunals Are Not the Answer. J Med Ethics (2016) 42(2):69–72. 10.1136/medethics-2015-102692 25887516

[60] Cooper RJ Wingfield J Bissell P. Ethical, Religious and Factual Beliefs About the Supply of Emergency Hormonal Contraception by UK Community Pharmacists. BMJ Sex Reprod Health (2008) 34(1):47–50. 10.1783/147118908783332122 18201407

[61] Maxwell C McKendrick J Hanlon C Penson P Fleming V. Exploring Pharmacists’ Views Surrounding Conscientious Objection to Abortion and Implications in Practice. Int J Pharm Pract (2021) 29(3):258–64. 10.1093/ijpp/riab009 33876827

[62] Voultsos P Zymvragou CE Raikos N. Perceptions and Experiences of Female Nurses When Confronted With Expressing a Conscientious Objection Towards End-of-Life Care in Greece. BMC Nurs (2023) 22:372. 10.1186/s12912-023-01555-8 37817234 PMC10563366

[63] Fleming V Frith L Luyben A Ramsayer B. Conscientious Objection to Participation in Abortion by Midwives and Nurses: A Systematic Review of Reasons. BMC Med Ethics (2018) 19(1):31. 10.1186/s12910-018-0268-3 29703258 PMC5923188

[64] Ramsayer B Fleming V. Conscience and Conscientious Objection: The Midwife’s Role in Abortion Services. Nurs Ethics (2020) 27(8):969733020928416. 10.1177/0969733020928416 32627664 PMC7575296

